# Clinical characteristics of smoking-related chronic pancreatitis

**DOI:** 10.3389/fcimb.2022.939910

**Published:** 2022-08-18

**Authors:** Lu Hao, Yu Liu, Zhi-Qi Dong, Jin-Hui Yi, Dan Wang, Lei Xin, Hong-Lei Guo, Lin He, Ya-Wei Bi, Jun-Tao Ji, Teng Wang, Ting-Ting Du, Jin-Huan Lin, Di Zhang, Xiang-Peng Zeng, Wen-Bin Zou, Hui Chen, Jun Pan, Zhuan Liao, Guo-Qiang Xu, Zhao-Shen Li, Liang-Hao Hu

**Affiliations:** ^1^ Department of Gastroenterology, First Affiliated Hospital, Zhejiang University School of Medicine, Hangzhou, China; ^2^ Department of Gastroenterology, Changhai Hospital, The Second Military Medical University, Shanghai, China; ^3^ Department of Gastroenterology and Hepatology, Jinling Hospital, Medical School of Nanjing University, Nanjing, China; ^4^ Department of Gastroenterology, Shanghai Fourth People’s Hospital, Tongji University School of Medicine, Shanghai, China; ^5^ Department of Gastroenterology and Endocrinology, 969th Hospital of People's Liberation Army (PLA), Hohhot, China; ^6^ Department of Gastroenterology and Hepatology, Chinese People's Liberation Army (PLA) General Hospital, Beijing, China; ^7^ Shanghai Guangming Middle School, Shanghai, China; ^8^ Department of Gastroenterology, 900th Hospital of Joint Logistics Support Force, Fuzhou, China

**Keywords:** chronic pancreatitis, smoking, drinking, etiology, natural course

## Abstract

**Objective:**

The pathogenesis of chronic pancreatitis (CP) is not completely clear. With further studies, smoking is toxic to the pancreas. This study classified smoking-related CP as a new etiology of CP and defined the cutoff of smoking.

**Design:**

Patients with CP admitted from January 2000 to December 2013 were included in the study. The characteristics were compared between smoking patients, drinking patients, and a group of patients who never smoke or drink (control group). The cumulative rates of steatorrhea, diabetes mellitus (DM), pancreatic pseudocyst (PPC), pancreatic stone, and biliary stricture after the onset of CP were calculated, respectively.

**Results:**

A total of 1,324 patients were included. Among them, 55 were smoking patients, 80 were drinking patients, and 1,189 were controls. The characteristics of smokers are different from the other two groups, especially in age at the onset and diagnosis of CP, initial manifestation, and type of pain. The development of DM (*P* = 0.011) and PPC (*P* = 0.033) was significantly more common and earlier in the smokers than in the other two groups. Steatorrhea also developed significantly more in the smokers than in the controls (*P* = 0.029). Smokers tend to delay the formation of pancreatic stones and steatorrhea.

**Conclusion:**

The clinical characteristics of smoking-related CP is different from CP of other etiologies. A new type of CP, smoking-related CP, was put forward. Smoking-related CP should be separated from idiopathic CP and defined as a new independent subtype of CP different from alcoholic CP or idiopathic CP.

## Introduction

The characteristic of chronic pancreatitis (CP) is gradual and irreversible damage of the pancreatic structure. Calcification of the pancreas, ductal calculus, stenosis and dilation of the pancreatic duct, and parenchymal atrophy were the morphologic changes of CP. Acute and chronic pancreatitis were considered as distinct entities as late as the Marseilles conference in 1984 (Singer et al., 1985). The criteria of alcoholic CP were defined by Lankisch PG et al. until 1995 (Lankisch et al., 1995). A recent study reported that 51.6% of CP patients were smokers (Han et al., 2018) who have a worse quality of life.

Because of its harmful role in CP, smoking continues to attract attention. It was reported as a clear risk factor for CP development in a dose-dependent manner (Talamini et al., 1999; Maisonneuve et al., 2005; Maisonneuve et al., 2006; Tolstrup et al., 2009; Yadav et al., 2009; Law et al., 2010; Yadav et al., 2010; Yadav and Whitcomb, 2010; Rebours et al., 2012; Sadr-Azodi et al., 2012) and has a toxic effect similar to alcohol. Smoking has a synergistic effect with alcohol, which accelerates the development of CP. Furthermore, once CP develops, smoking can promote the formation of complications such as exocrine insufficiency, pancreatic calcifications, and pseudocysts, suggesting that smoking may accelerate the progression of CP (Bernard et al., 1992; Cavallini et al., 1994; Hartwig et al., 2000; Cote et al., 2011; Luaces-Regueira et al., 2014; Greer et al., 2015; Sankaran et al., 2015; Ahmed Ali et al., 2016; Lee et al., 2016; Setiawan et al., 2016).

Wittel *et al.* demonstrated that high-dose tobacco exposure in rats led to the damage of the pancreas with CP features at the molecular levels. Chowdhury *et al.* showed that, in the pancreas of rats, nicotine was significantly accumulated, suggesting that nicotine may play an active role in pancreatic inflammation (Chowdhury et al., 1993; Chowdhury et al., 2002; Wittel et al., 2006; Wassef et al., 2014; Han et al., 2016). With the in-depth research and evidence during the past decades, we assume that smoking-related CP may be an independent subgroup of CP.

This study was based on a retrospective prospective cohort of 2,153 CP patients, who were followed up after the onset with a long duration. We aimed to assess the epidemiological features, natural course, and complications as well as compare them between smokers, alcoholics, and without tobacco or alcohol users.

## Materials and methods

### Patients and database

The database of CP in our hospital (version 2.1, YINMA Information Technology Inc., Shanghai, China) was established in 2005, which has been reported in several studies on CP (Li et al., 2014; Xin et al., 2014; Sun et al., 2015; Li et al., 2016; Pan et al., 2016; Hao et al., 2017; Hao et al., 2017; Hao et al., 2017). Information including tobacco and alcohol consumption, course of CP, demographic data (age, sex, birthplace, *etc.*), history of other diseases, medical history, laboratory and imaging findings, family history of pancreatic diseases and diabetes mellitus (DM), and treatment strategy were documented in detail.

Patients in the database system were called for clinical checkups. In addition to follow-up due to discomfort associated with CP, each patient is recalled regularly (at least annually) for clinical examinations. Computed tomography, ultrasound, or magnetic resonance imaging was chosen as the assessment method during each visit. An assessment of the patients who have not returned to our center for each revisit or by telephone enquiry has been added to the database. In December 2013, we contacted all patients included in the database for a final assessment, except for patients who died or were lost to follow-up. The follow-up period was defined as the time from the onset of CP to death, the last personal contact, or end of follow-up (December 2013), whichever comes first.

The study was approved by the Ethics Committee of Changhai Hospital, The Second Military Medical University, Shanghai, China. All participating patients received written informed consent. All diagnosis and treatment methods were carried out in accordance with existing guidelines (Maydeo et al., 2007; Schreyer et al., 2014; Ito et al., 2016; Dumonceau et al., 2019).

### Definitions

CP can be diagnosed when one of the following conditions is established: (1) pancreatic ductal changes (according to the Cambridge classification system, moderate or marked disease), (2) pancreatic calcification appearance, (3) characteristics of CP in endoscopic ultrasound, (4) abnormal results of pancreatic endocrine or exocrine function, or (5) histological proof of CP as described by the Asia-Pacific consensus (Tandon et al., 2002). The onset of CP was considered when the first symptom relevant to CP occurred, such as acute pancreatitis attack, recurrent pancreatic pain, DM, steatorrhea, chronic pancreatic pain, or asymptomatic patients diagnosed of CP in the course of physical examinations.

According to the etiologies of CP that we have known, hereditary CP refers to two first-degree relatives or three or more second-degree relatives in two or more generations with recurrent acute pancreatitis and/or CP without any precipitating factors (Howes et al., 2004). The abnormal anatomy of the pancreatic duct refers to anomalous pancreaticobiliary junction and pancreas divisum (Lu, 1998). For patients who have a history of abdominal injury with imaging confirmation of pancreatic injury and subsequent ductal dilation, this was defined as post-traumatic CP. When the blood triglycerides are >1,000 mg/dl, hyperlipidemia is considered as an etiology (Yadav and Pitchumoni, 2003). Alcoholic CP was considered when the alcohol consumption exceeded 80 g/day for men or 60 g/day for women for at least 2 years without other causes (Lankisch et al., 1995). In the present study, patients were defined as smoking-related CP when they smoke for more than 30 pack-years. As the toxic effect has already impaired the pancreas, patients who met the criteria of alcoholic intake, whether they have quit drinking or not, were all regarded as alcoholic CP. Similarly, patients who met the criteria of tobacco intake, whether they have quit smoking or not, were regarded as smoking-related CP. When none of the above-mentioned causes was found, idiopathic CP was considered.

To explore the proper cutoff value of smoking consumption to define smoking-related CP, the distribution of cigarette consumption was calculated in the CP patients. In all the smokers, it peaked at 30–35 pack-year and gradually declined (**Supplementary Figure S1A**). After the exclusion of patients who ever drunk, it peaked at 20–25 pack-years (**Supplementary Figure S1B**). A comparison of complications at the diagnosis of CP between different cutoffs was also calculated (**Table 1**). For patients who smoke 10 to 30 pack-years, the development of pancreatic pseudocyst (PPC) is significantly different from that of the drinking patients and controls. For patients who smoke 20 to 30 pack-years, the development of steatorrhea is significantly different from that of the controls. Thus, the cutoff for smoking-related CP of 30 pack-years was selected according to the aforementioned findings and previous studies (Park et al., 2014; Tammemagi et al., 2014).

**Table 1:** Comparison between different cutoffs of smoking.

Cutoff (pack-year)	Smoker, *n*	Drinker, *n*	Control, *n*	Stone		DM		Steatorrhea		Biliary stricture		PPC	
				*P* ^a^	*P* ^b^	*P* ^a^	*P* ^b^	*P* ^a^	*P* ^b^	*P* ^a^	*P* ^b^	*P* ^a^	*P* ^b^
40	15	83	1,189	0.584	0.309	0.831	0.744	0.450	0.316	0.733	0.885	0.313	0.288
35	26	83	1,189	0.996	0.520	0.771	0.690	0.290	0.143	0.513	0.696	0.513	0.461
30	55	83	1,189	0.285	0.648	0.896	0.090	0.140	0.029	0.385	0.622	0.009	0.017
20	90	83	1,189	0.349	0.604	0.459	0.273	0.087	0.011	0.102	0.083	0.010	0.035
15	114	83	1,189	0.238	0.361	0.432	0.309	0.256	0.073	0.174	0.184	0.008	0.025
10	133	83	1,189	0.266	0.374	0.359	0.296	0.363	0.112	0.127	0.072	0.030	0.153
5	147	83	1,189	0.212	0.310	0.319	0.241	0.514	0.238	0.129	0.080	0.055	0.307

DM, diabetes mellitus; PPC, pancreatic pseudocyst.

a Comparison between the smoker and the drinker groups.

b Comparison between the smoker and the control groups.

**Figure 1:**
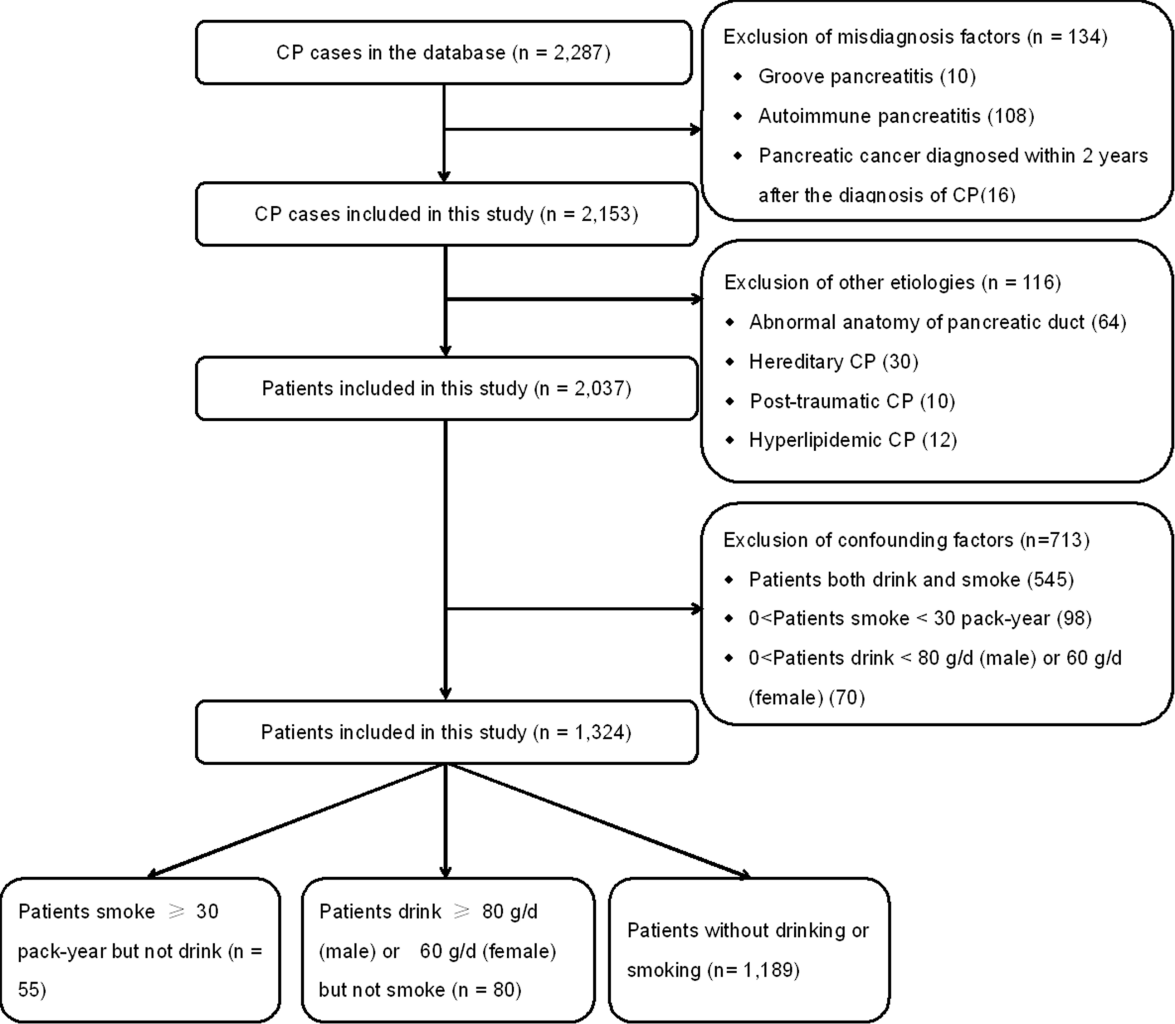
Flow diagram of the patients’ enrollment and study design.

Patients with the following features were excluded (**Figure 1**): patients diagnosed with pancreatic cancer within 2 years after the onset of CP (Li et al., 2014), autoimmune pancreatitis, and groove pancreatitis (Malde et al., 2011). Patients with other etiologies (including hereditary, hyperlipidemic, abnormal anatomy of pancreatic duct, and post-traumatic) were also excluded. In order to exclude confounding factors, patients who both drink and smoke, patients who smoke <30 pack-years, and patients who drink <80 g/day (men) or 60 g/day (women) were further excluded.

Thus, patients with 30 or more pack-years of smoking history were defined as the smoking patients. Patients with alcohol consumption that exceeded 80 g/day for men or 60 g/day for women for at least 2 years were defined as the drinking patients. Idiopathic CP patients who never smoke or drink were assigned to the controls.

### Statistical analysis

Continuous variables are expressed as mean ± standard deviation. Comparisons between smokers and drinkers and between smokers and controls were made using an unpaired two-tailed *t*-test or Mann–Whitney test. The categorical variables were compared using *χ* ^2^ test or Fisher’s exact test. The Kaplan–Meier method was used to calculate the cumulative rate of DM, steatorrhea, pancreatic stones, PPC, and biliary stricture after CP onset. Log-rank test was used to analyze between groups for any significant differences.

## Results

### General characteristics of CP patients

After the exclusion of 250 patients, a cohort of 2,037 CP patients was included, which are listed in **Figure 1**. In the present study, 545 patients who both drink and smoke were excluded to reduce the confounding bias. Moreover, 98 patients who smoke <30 pack-years and 70 patients who drink <80 g/day (men) or 60 g/day (women) were also further excluded. Finally, a cohort of 1,324 patients was enrolled.

As shown in **Table 2** show, the general features of these CP patients are listed. The median follow-up duration was 7.6 (range, 0.0–53.2) years. In the 55 smokers, the median follow-up duration was 5.3 (range, 0.2–39.0) years. Among the 80 drinkers, the median follow-up duration was 11.0 (range, 1.5–43.2) years, while in the 1,189 controls, the median follow-up time was 7.4 (range, 0.0–53.2) years. Age at onset of CP, initial manifestations, age at diagnosis of CP, age at pancreatic stone, DM, biliary stricture diagnosis, and the pain type were significantly different between smokers and drinkers (all *P <*0.05). Gender, age at the onset and diagnosis of CP, body mass index, initial manifestations, age at stone and steatorrhea, PPC, and type of pain were significantly different between smokers and controls (all *P <*0.05).

**Table 2:** General characteristics of 1,324 patients with CP.

Items	Smoker *N* = 55	Drinker *N* = 80	Control *N* = 1,189	*P* ^a^	*P* ^b^
Male sex	55 (100.0%)	80 (100.0%)	594 (50.0%)	–	<0.001
Age at the onset of CP, years^c^	49.669 ± 11.064	37.879 ± 12.131	37.106 ± 18.770	<0.001	<0.001
Age at the diagnosis of CP, years^c^	55.120 ± 7.972	45.198 ± 10.342	41.369 ± 17.763	<0.001	<0.001
Adolescent	0	2 (2.5%)	231 (19.3%)	0.237	<0.001
Body mass index^c^	23.096 ± 4.501	21.515 ± 4.149	20.474 ± 3.407	0.063	<0.001
Initial manifestations				0.001	<0.001
Abdominal pain	39 (70.9%)	70 (87.5%)	985 (82.8%)		
Endocrine/exocrine dysfunction	4 (7.3%)	8 (10.0%)	133 (11.2%)		
Others	12 (21.8%)	2 (2.5%)	71 (6.0%)		
Pancreatic stones^d^	42 (76.4%)	68 (85.0%)	828 (69.6%)	0.204	0.288
Age at pancreatic stone diagnosis^c^	55.038 ± 7.808	46.815 ± 10.842	38.074 ± 17.505	<0.001	<0.001
Time between onset and pancreatic stone^c^	6.182 ± 7.338	8.562 ± 8.350	6.097 ± 6.766	0.090	0.928
DM	18 (32.7%)	36 (45.0%)	297 (25.0%)	0.153	0.196
Age at diabetes^c^	50.276 ± 9.803	44.599 ± 9.687	46.378 ± 13.097	0.048	0.216
Time between onset and DM^c^	4.484 ± 11.063	8.449 ± 6.846	4.438 ± 7.438	0.111	0.980
Steatorrhea	16 (29.1%)	23 (28.8%)	232 (19.5%)	0.966	0.082
Age at steatorrhea^c^	50.252 ± 11.677	43.108 ± 9.753	41.389 ± 14.292	0.064	0.016
Time between onset and steatorrhea^c^	3.707 ± 6.843	5.792 ± 6.405	4.848 ± 8.334	0.373	0.593
Biliary stricture	9 (16.4%)	13 (16.3%)	177 (14.9%)	0.986	0.764
Age at CBD stenosis^c^	56.128 ± 10.122	46.259 ± 11.043	53.895 ± 14.440	0.046	0.648
Time between onset and CBD stenosis^c^	3.721 ± 4.749	6.889 ± 9.722	5.136 ± 8.949	0.378	0.639
Pancreatic pseudocyst	13 (23.6%)	10 (12.5%)	166 (14.0%)	0.091	0.046
Age at pseudocyst^c^	49.203 ± 8.650	48.628 ± 9.264	44.110 ± 17.263	0.881	0.081
Time between onset and pseudocyst formation^c^	4.543 ± 5.925	7.383 ± 8.817	3.004 ± 5.467	0.366	0.381
Pancreatic cancer	0	0	18 (1.5%)	–	0.358
Death	0	3 (3.8%)	57 (4.8%)	0.146	0.096
Morphology of MPD				0.619	0.376
Pancreatic stone alone	21 (38.2%)	31 (38.8%)	339 (28.5%)		
MPD stenosis alone	18 (32.7%)	19 (23.8%)	384 (32.3%)		
MPD stenosis and stone	12 (21.8%)	24 (30.0%)	342 (28.8%)		
Complex pathologic changes	4 (7.3%)	6 (7.2%)	124 (10.4%)		
Type of pain				0.041	0.018
Recurrent acute pancreatitis	17 (30.9%)	41 (51.3%)	346 (29.1%)		
Recurrent pain	8 (14.5%)	14 (17.5%)	400 (33.6%)		
Recurrent acute pancreatitis and pain	17 (30.9%)	16 (20.0%)	296 (24.9%)		
Chronic pain	5 (9.1%)	1 (1.3%)	54 (4.5%)		
Without pain	8 (14.5%)	8 (10.0%)	93 (7.8%)		
Severe acute pancreatitis	2 (3.6%)	5 (6.3%)	36 (3.0%)	0.501	0.798
Successful drainage^e^	34 (61.8%)	53 (66.3%)	798 (67.1%)	0.597	0.414
Overall treatment				0.689	0.621
Endotherapy alone	39 (70.9%)	57 (71.3%)	753 (63.3%)		
Surgery alone	6 (10.9%)	5 (6.3%)	170 (14.3%)		
Both endotherapy and surgery	5 (9.1%)	11 (13.8%)	102 (8.6%)		
Conservative treatment	5 (9.1%)	7 (8.8%)	164 (13.8%)		
DM in first-/second-/third-degree relatives	5 (9.1%)	5 (6.3%)	49 (4.1%)	0.536	0.077
Pancreatic diseases in first-/second-/third-degree relatives (excluding hereditary CP)	1 (1.8%)	0	14 (1.2%)	0.226	0.670

CP, chronic pancreatitis; DM, diabetes mellitus; ICP, idiopathic chronic pancreatitis; ACP, alcoholic chronic pancreatitis; HCP, hereditary chronic pancreatitis.

a Comparison between the smoker and the drinker groups.

b Comparison between the smoker and the control groups.

c Mean ± SD.

d Pancreatic calcifications were also regarded as stones that are located in a branch of the pancreatic duct or ductulus.

e Patients with successful main pancreatic duct (MPD) drainage are those whose CP was established after ERCP or pancreatic surgery or those who underwent successful MPD drainage during administration when CP diagnosis was established.

### Cumulative rates in smokers, drinkers, and control

#### Cumulative rates of DM

**Figure 2:**
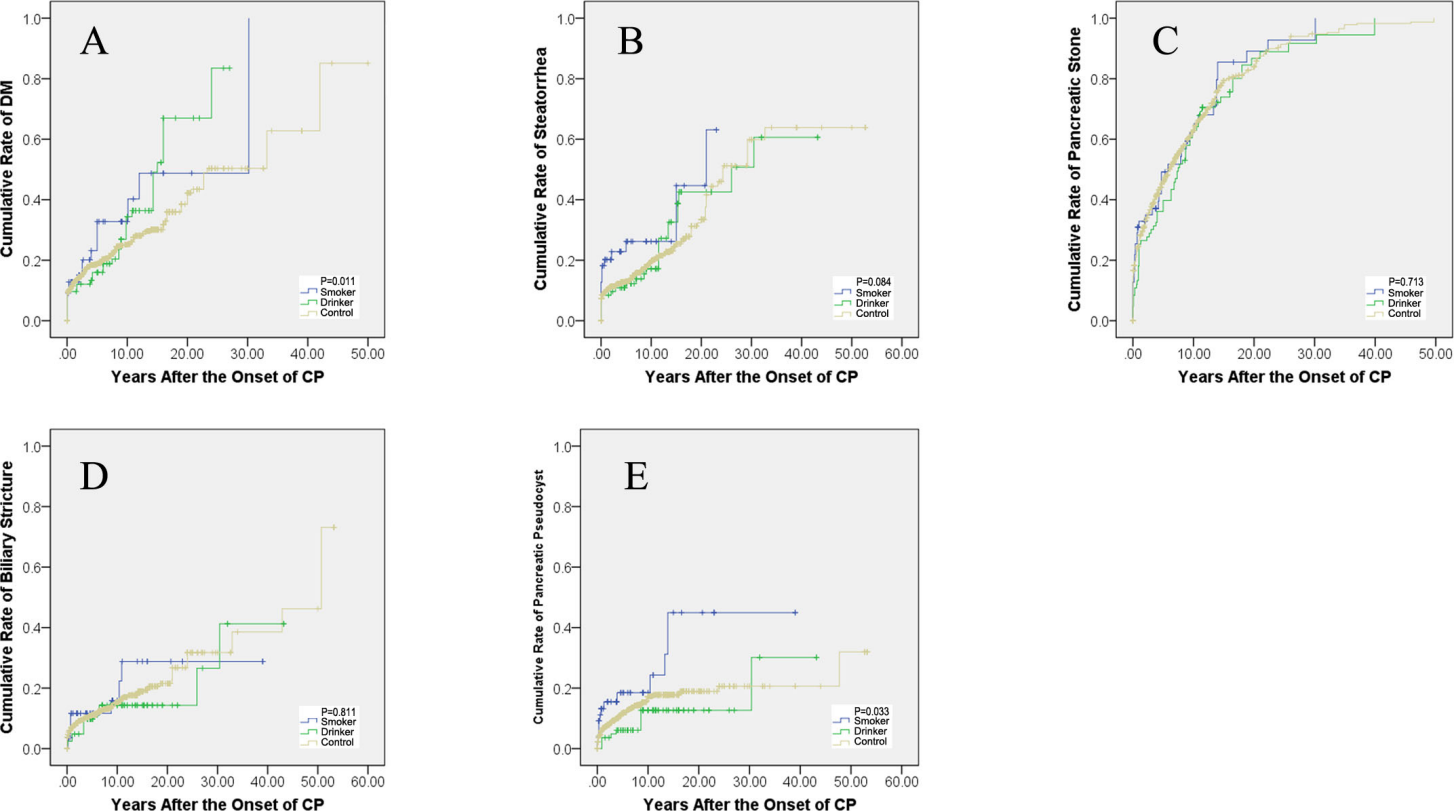
Cumulative rates after the onset of CP. **(A)** The cumulative rates of diabetes mellitus. **(B)** Cumulative rates of steatorrhea. **(C)** Cumulative rates of pancreatic stone. **(D)** Cumulative rates of biliary stricture. **(E)** Cumulative rates of pancreatic pseudocysts. CP, chronic pancreatitis; DM, diabetes mellitus.

DM developed in 26.5% (351/1,324) of the included patients in this study. The rate was 32.7% (18/55) in smokers, 45.0% (36/80) in drinkers, and 25.0% (297/1,189) in the control patients. DM was diagnosed in 10, 11, and 14 patients at 3, 5, and 10 years in the smokers after the onset of CP, the cumulative rates of which were 18.2% [95% confidence interval (CI): 12.0–24.4%], 20.0% (95% CI: 12.6–27.4%), and 25.5% (95% CI: 18.0–32.9%), and in 10, 13, and 24 patients in the drinking patients, the cumulative rates of which were 12.5% (95% CI: 8.9–16.1%), 16.3% (95% CI: 12.1–20.4%), and 30.0% (95% CI: 24.0–36.0%), while in 194, 210, and 250 patients in the controls, the cumulative rates were 16.3% (95% CI: 15.2–17.4%), 17.7% (95% CI: 16.5–18.8%), and 21.0% (95% CI: 19.6–22.5%), respectively. The rates of DM after the onset of CP showed a significant difference between the three groups (*P* = 0.011, **Figure 2A**).

#### Cumulative rates of steatorrhea

Steatorrhea was diagnosed in 20.5% (271/1,324) of the included patients in this study. The rate was 29.1% (16/55) in smokers, 28.8% (23/80) in drinkers, and 19.5% (232/1,189) in the control patients. Steatorrhea developed in 12, 13, and 13 patients at the third, fifth, and 10th year in the smoking patients after the onset of CP, the cumulative rates of which were 21.8% (95% CI: 16.0–27.6%), 23.6% (95% CI: 17.4–30.0%), and 23.6% (95% CI: 17.4–30.0%), and in 9, 10, and 12 patients in the drinking patients, the cumulative rates of which were 11.3% (95% CI: 7.8–14.7%), 12.5% (95% CI: 8.8–16.2%), and 15.0% (95% CI: 10.8–19.2%), while in 138, 149, and 189 patients in the controls, the cumulative rates were 11.6% (95% CI: 10.6–12.6%), 12.5% (95% CI: 11.6–13.5%), and 15.9% (95% CI: 14.5–17.3%), respectively. The rate of steatorrhea after the onset of CP showed no significant difference between the three groups (*P* = 0.084, **Figure 2B**).

#### Cumulative rates of pancreatic stone

Pancreatic stone was diagnosed in 70.8% (938/1,324) of the included patients in this study. The rate was 76.4% (42/55) in smokers, 85.0% (68/80) in drinkers, and 69.6% (828/1,189) in the control patients. Pancreatic stone was diagnosed in 19, 25, and 30 patients at the third, fifth, and 10th year in the smoking patients after the onset of CP, the cumulative rates of which were 34.5% (95% CI: 28.2–40.9%), 45.5% (95% CI: 38.4–52.5%), and 54.5% (95% CI: 47.3–61.8%), and in 25, 33, and 51 patients in the drinking patients, the cumulative rates of which were 31.3% (95% CI: 26.2–36.3%), 41.3% (95% CI: 35.9–46.6%), and 63.8% (95% CI: 58.4–69.1%), while in 438, 524, and 672 patients in the controls, the cumulative rates were 36.8% (95% CI: 35.5–38.2%), 44.1% (95% CI: 42.6–45.5%), and 56.5% (95% CI: 55.0–58.1%), respectively. The rates of pancreatic stone after the onset of CP showed no significant difference between the three groups (*P* = 0.713, **Figure 2C**).

#### Cumulative rates of biliary stricture

Biliary stricture was diagnosed in 15.0% (199/1,324) of the included patients in this study. The rates were 16.4% (9/55) in smokers, 16.3% (13/80) in drinkers, and 14.9% (177/1,189) in the control patients. Biliary stricture developed in six, six, and seven patients at the third, fifth and 10th year in the smoking patients after the onset of CP, the cumulative rates of which were 10.9% (95% CI: 6.5–15.3%), 10.9% (95% CI: 6.5–15.3%), and 12.7% (95% CI: 6.9–18.5%), and in four, eight, and 11 patients in the drinking patients, the cumulative rates of which were 5.0% (95% CI: 2.6–7.4%), 10.0% (95% CI: 6.7–13.3%), and 13.8% (95% CI: 9.6–17.9%), while in 108, 124, and 149 patients in the controls, the cumulative rates were 9.1% (95% CI: 8.2–10.0%), 10.4% (95% CI: 9.4–11.4%), and 12.5% (95% CI: 11.3–13.7%), respectively. The rates of biliary stricture after the onset of CP showed no significant difference between the three groups (*P* = 0.811, **Figure 2D**).

#### Cumulative rates of PPC

PPC developed in 14.3% (189/1,324) of the included patients in this study. The rate was 23.6% (13/55) in smokers, 12.5% (10/80) in drinkers, and 14.0% (166/1,189) in the control patients. PPC developed in eight, nine, and nine patients at the third, fifth, and 10th year in the smoking patients after the onset of CP, the cumulative rates of which were 14.5% (95% CI: 9.5–19.5%), 16.4% (95% CI: 10.8–21.9%), and 16.4% (95% CI: 10.8–21.9%), and in four, five, and 11 patients in the drinking patients, the cumulative rates of which were 5.0% (95% CI: 2.6–7.4%), 6.3% (95% CI: 3.6–8.9%), and 11.3% (95% CI: 7.1–15.4%), while in 105, 126, and 160 patients in the controls, the cumulative rates were 8.8% (95% CI: 7.9–9.7%), 10.6% (95% CI: 9.6–11.6%), and 13.5% (95% CI: 12.2–14.7%), respectively. The rates of PPC after the onset of CP showed a significant difference between the three groups (*P* = 0.033, **Figure 2E**).

## Discussion

This is a research about the natural course of CP smokers and drinkers based on a large sample of CP patients. A new type of CP, smoking-related CP, was put forward. In this study, the characteristics of CP, initial performance, natural course of the disease, and complications were accessed. The study included patients who only smoke or only drink, which ruled out the combined effects of tobacco and alcohol.

Age at the onset of CP and age at the diagnosis of CP were significantly different in these three groups. Smokers tended to have a later onset and diagnosis of CP than the drinkers and controls (both *P* < 0.001). However, smoking was reported to hasten the age of first diagnosis in a previous study (Maisonneuve et al., 2005), which is contradictory to the present finding. It may be due to the fact that only patients who smoke >30 pack-years were included in the present study, which needed several years to reach the criteria. A significant difference was observed in the initial manifestations in these three groups (*P* = 0.001 and *P* < 0.001 respectively). Smokers are less likely to have abdominal pain or endocrine/exocrine dysfunction at the onset of CP. It can also be proved by the type of pain (*P* = 0.041 and *P* = 0.018 respectively). Patients without pain occupied a larger proportion in the smokers. At the diagnosis of CP, steatorrhea developed more in the smoking patients than in the controls (*P* = 0.029), PPC developed more in the smoking groups than in the other two groups (*P* = 0.009 and *P* = 0.017, respectively). After the follow-up, DM developed more and faster in the smoking patients than in the drinkers and controls (*P* = 0.011); PPC developed more and faster as well in the smoking patients than in the drinking patients and controls (*P* = 0.033). Smokers tend to have a later formation of pancreatic stones than the other two groups (all *P* <0.001) and delayed occurrence of steatorrhea than controls (P=0.016).

Smoking was reported as a definite risk factor for CP, accelerating disease progression both from the onset of CP and within CP in numerous studies (Cote et al., 2011; Rebours et al., 2012; Sadr-Azodi et al., 2012; Ahmed Ali et al., 2016; Setiawan et al., 2016). Cigarette smoking is reported to accelerate pancreatic calcification and functional impairment (Talamini et al., 1996; Maisonneuve et al., 2005; Yadav et al., 2009; Law et al., 2010), which is in accordance with the present study. Cigarette smoking will also enhance ethanol-induced pancreatic injury (Hartwig et al., 2000). In the present study, patients who only smoke or only drink were included, which excluded the combined effect of tobacco and alcohol. According to the results of the comparison, the toxic impairment of pancreas caused by tobacco and alcohol is not exactly the same. Smoking may accelerate the damage of pancreatic endocrine and exocrine function as well as development of PPCs. Accordingly, less pain was observed in the smoking patients. Thus, cigarette smoking may be an independent etiology of CP. According to our present study, patients with a 30 or more pack-year smoking history in the absence of other CP etiologies should be identified as smoking-related CP. Smoking-related CP is a unique subgroup of CP which is different from other types of CP with other etiologies. Thus, smoking-related CP should be separated from idiopathic CP.

The identification of modifiable etiology provides evidence for guiding clinical practice and patient education—for example, lifestyle modifications such as tobacco abstinence, as recommended for CP patients, have been further confirmed by identifying smoking as an etiology of CP. Patients with smoking-related CP should be screened more frequently for DM, steatorrhea, and PPC. Once DM or steatorrhea occurred, insulin or pancreatic enzyme replacement therapy should be performed.

There are some limitations in our research. First, the data was collected retrospectively from 2000 to 2004, which may lead to recall bias. However, patients admitted to our hospital before and after January 2005 showed no significant difference in clinical characteristics. Based on the statistical analysis presented above, the recall bias has little effect on the results of the study. Second, 149 patients diagnosed as CP have a follow-up time of less than 2 years after the diagnosis. Among the 149 patients, several of them with pancreatic cancer may have been misdiagnosed as CP. However, given the relatively large sample size of the study, these limitations have a little effect on the results. Third, as tobacco and alcohol are dose-dependent factors for CP development, toxins accumulation may cost years. The smoking and drinking patients are older than the controls. Adjustment of ages may be needed in a further study. Furthermore, the number of patients included in the smoking and drinking group is relatively small, which may increase the risk of selection and data collection bias and limit the possibility of making definite conclusions. A further study in a large sample prospective cohort is needed.

## Conclusion

In conclusion, there is a really different clinical course of CP caused by smoking from that caused by other etiologies. Smoking may accelerate the damage of pancreatic endocrine and exocrine function as well as development of PPCs. Therefore, less pain was observed. A new type of CP, smoking-related CP, was put forward. Smoking-related CP may be separated from idiopathic CP and thus defined as a new independent subtype of CP different from alcoholic CP or idiopathic CP. Further studies focused on smoking-related CP are needed.

## Data availability statement

The raw data supporting the conclusions of this article will be made available by the authors without undue reservation.

## Ethics statement

The studies involving human participants were reviewed and approved by the Ethics Committee of Changhai Hospital, The Second Military Medical University, Shanghai, China. Written informed consent to participate in this study was provided by the participants’ legal guardian/next of kin.

## Author contributions

LHao and YL participated in the acquisition, analysis, and interpretation of data as well as in manuscript drafting. Z-QD, J-HY, H-LG, DW, LHe, Y-WB, J-TJ, LX, TW, T-TD, J-HL, DZ, X-PZ, W-BZ, HC, JP, and ZL participated in data acquisition and manuscript drafting. G-QX, Z-SL, and L-HH contributed to the conception, design, and data interpretation as well as revised the manuscript for important intellectual content. All authors contributed to the article and approved the submitted version.

## Funding

This study was supported by the National Natural Science Foundation of China [grant nos. 82070664 (L-HH), 82000608 (LHao), and 81900590 (DW)], Shanghai Science and Technology Innovation Action Plan [grant no. 19DZ2201900 (L-HH)], Shanghai Shuguang Program [grant no. 20SG36 (L-HH)], Shanghai Excellent Young Medical Talents Program [grant no. 2018YQ49 (LX)], Medicine Guidance Project of Shanghai [grant no. 17411971500 (LX)], and Shanghai Sailing Program [grant no. 19YF1446800 (DW)].

## Acknowledgment

The authors gratefully acknowledge Dr. Xi Jin, Department of Gastroenterology, First Affiliated Hospital, Zhejiang University School of Medicine, Hangzhou, China, for his critical review and recommendation.

## Conflict of interest

The authors declare that the research was conducted in the absence of any commercial or financial relationships that could be construed as a potential conflict of interest.

## Publisher’s note

All claims expressed in this article are solely those of the authors and do not necessarily represent those of their affiliated organizations, or those of the publisher, the editors and the reviewers. Any product that may be evaluated in this article, or claim that may be made by its manufacturer, is not guaranteed or endorsed by the publisher.

## Supplementary material

Supplementary Figure 1 Distribution of cigarette consumption in CP patients. **(A)** Distribution of cigarette consumption in all CP patients. **(B)** Distribution of cigarette consumption in non-drinkers of CP. CP, chronic pancreatitis.

## Abbreviations

AIP, autoimmune pancreatitis; CP, chronic pancreatitis; CT, computed tomography; DM, diabetes mellitus; ERCP, endoscopic retrograde cholangiopancreatography; ESWL, extracorporeal shockwave lithotripsy; GP, groove pancreatitis; MPD, main pancreatic duct; MRI, magnetic resonance imaging; PPC, pancreatic pseudocyst; SD, standard deviation.
