# Supplementary figures and images for: Clinical characteristics of smoking-related chronic pancreatitis
Source: Front Cell Infect Microbiol. 2022 Aug 18;12:939910. doi: 10.3389/fcimb.2022.939910 (PMC9433580; doi:10.3389/fcimb.2022.939910)

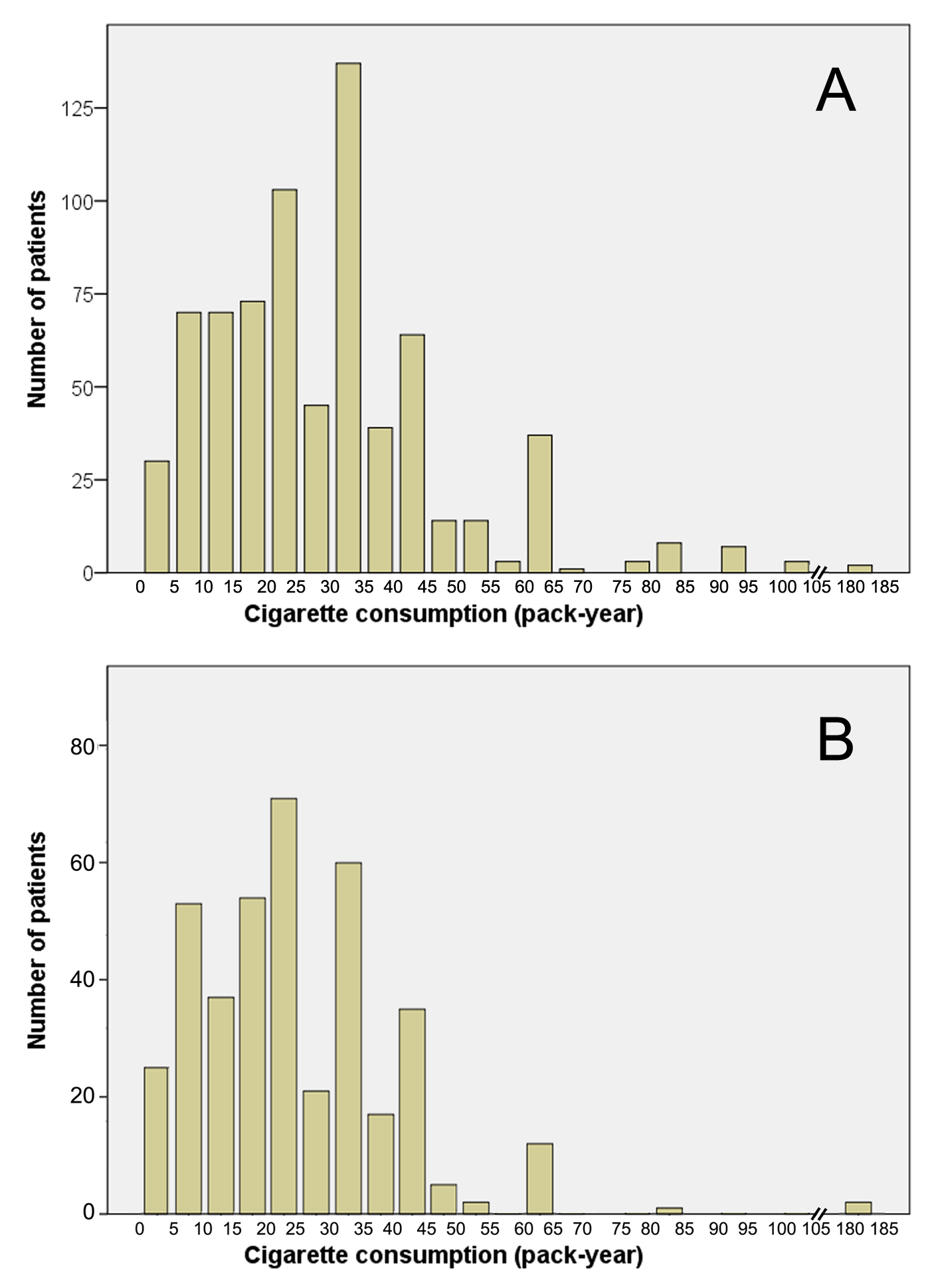

Supplement: Supplementary Figure 1 — Distribution of cigarette consumption in CP patients. (A) Distribution of cigarette consumption in all CP patients. (B) Distribution of cigarette consumption in non-drinkers of CP. CP, chronic pancreatitis. [file Image_1.tif]
